# The Phytotoxin Coronatine Induces Abscission-Related Gene Expression and Boll Ripening during Defoliation of Cotton

**DOI:** 10.1371/journal.pone.0097652

**Published:** 2014-05-20

**Authors:** Mingwei Du, Yi Li, Xiaoli Tian, Liusheng Duan, Mingcai Zhang, Weiming Tan, Dongyong Xu, Zhaohu Li

**Affiliations:** 1 State Key Laboratory of Plant Physiology and Biochemistry, Engineering Research Center of Plant Growth Regulator, Ministry of Education, College of Agronomy and Biotechnology, China Agricultural University, Beijing, China; 2 Hebei Provincial Engineering Research Center of Cotton Seed, Hejian, Hebei, China; New Mexico State University, United States of America

## Abstract

Defoliants can increase machine harvest efficiency of cotton (*Gossypium hirusutum* L.), prevent lodging and reduce the time from defoliation to harvest. Coronatine (COR) is a chlorosis-inducing non-host-specific phytotoxin that induces leaf and/or fruit abscission in some crops. The present study investigates how COR might induce cotton leaf abscission by modulating genes involved in cell wall hydrolases and ACC (ethylene precursor) in various cotton tissues. The effects of COR on cotton boll ripening, seedcotton yield, and seed development were also studied. After 14 d of treatment with COR, cells within the leaf abscission zone (AZ) showed marked differentiation. Elevated transcripts of *GhCEL1*, *GhPG* and *GhACS* were observed in the AZs treated with COR and Thidiazuron (TDZ). The relative expression of *GhCEL1* and *GhACS* in TDZ treated plants was approximately twice that in plants treated with COR for 12 h. However, only *GhACS* expression increased in leaf blade and petiole. There was a continuous increase in the activity of hydrolytic enzymes such as cellulase (CEL) and polygalacturonase (PG), and ACC accumulation in AZs following COR and TDZ treatments, but there was greater increase in ACC activity of COR treated boll crust, indicating that COR had greater ripening effect than TDZ. Coronatine significantly enhanced boll opening without affecting boll weight, lint percentage and seed quality. Therefore, COR can be a potential cotton defoliant with different physiological mechanism of action from the currently used TDZ.

## Introduction

Cotton is an important commercial crop worldwide, and serves as a significant source of fiber, feed, foodstuff, oil and biofuel [1]. Defoliation or leaf abscission is induced in cotton as a natural physiological process which usually is inadequate or not timely enough for a complete mechanical harvest of cotton. Therefore, defoliation before harvest is often induced by managing the plants so that senescence, abscission (separation) layer development and leaf drop are encouraged [2], [3]. The ultimate goal of defoliants is to facilitate mechanical harvest, reduce trash and protect fiber and seed quality from weathering and staining by allowing earlier harvest [4]. Another benefit is the reduced moisture content in the raw fibers and seed which is essential for storage of seedcotton.

Selection of appropriate abscission chemicals is one of the critical decisions in cotton production. Herbicidal or hormonal defoliants, such as dimethipin and thidiazuron, are widely used in many cotton producing areas [5]. Dimethipin is a plant growth regulator used as a harvest aid on a variety of crops [6]. It causes leaf cells to slowly lose water and generates ethylene within plants. Dimethipin is considered a contact-type defoliant, whereas thidiazuron has growth-regulator properties and moves through the plant [7]. Thidiazuron increases the concentration of ethylene relative to auxin in leaf petioles and results in the activation of the leaf abscission layer [8], [9]. However, these types of defoliants induce drastic leaf abscission which inhibits timely transport of nutrients from leaves to cotton bolls. Also, these defoliants do not directly influence boll ripening and must be applied in combination with ethephon, a boll opener, to provide satisfactory defoliation and boll opening [5]. An abscission chemical with improved defoliation and boll opening properties is needed for cotton harvest practices.

Coronatine (COR) is a chlorosis-inducing non-host-specific phytotoxin produced by several members in the *Pseudomonas syringae* group of pathovars [10], [11]. It induces inhibition of root elongation, senescence, production of defense-related protease inhibitors, and resistance to abiotic stresses [12]–[16]. COR also induces growth regulator-like effects such as hypertrophy and stimulation of ethylene production and tendril coiling [17]–[20]. In addition, COR has been reported to be a structural and functional analog of jasmonic acid and methyl jasmonate, which are important plant growth substances in octadecanoid signaling [21]–[23]. Components of the octadecanoid pathway have been shown to affect the regulation of wounding [24], fruit ripening [25], and abscission [26]. External application of methyl jasmonate and COR likely induced abscission by stimulating levels of ethylene when applied to the entire citrus (*Citrus sinensis*) tree canopy [27], [28]. However, the ability of COR to cause leaf abscission in cotton is unclear.

Abscission is the main process that involves structural, biochemical, and molecular changes resulting in the detachment of plant organs, including leaves, flowers and fruits [29], [30]. Abscission occurs at predetermined sites referred to as abscission zones, which consist of a few layers of small, densely packed cells that respond in different ways from neighbouring cells to the same hormonal or environmental cues [31], [32]. Knowledge of mechanisms involved in abscission of leaves or other organs is essential to develop strategies to control them and improve harvesting practices or unwanted crop loss in fruit crops [33]. Once abscission is initiated, cells in the abscission zone begin to enlarge, followed by increased expression of genes and the activities of cell wall-degrading enzymes such as β-1, 4-glucanase or cellulase (CEL) and polygalacturonase (PG) [32], [34]–[36]. As a result, the middle lamellae of abscission zone cells dissolve and, ultimately, the organ abscises.

Ethylene plays a primary role in accelerating leaf abscission and fruit ripening [37]–[40]. The conversions of S-AdoMet (SAM) to 1-aminocyclopropane-1-carboxylic acid (ACC, metabolic precursor of ethylene) is the rate-limiting step in ethylene biosynthesis, and is catalysed by ACC synthese (ACS) [39], [41]. The observations that expression of the ACS genes is highly regulated by a variety of signals and that active ACC synthase is labile and present at low levels suggest that ethylene biosynthesis is tightly controlled [41]. Both positive and negative feedback regulation of ethylene biosynthesis have been reported in different plant species [30], [31], [42]–[44]. Most studies addressing ACS regulation have focused on ACS gene expression in response to various endogenous cues and environmental stimuli [31], [41], [42]. In an attempt to understand how responses to COR operate, some physiological- and transcriptional- level responses of cotton to the application of COR need further study.

The purpose of this study was first to investigate the possible roles of COR during cotton leaf abscission compared with using TDZ or water (control). In the present work, the phenotypic and anatomical changes in leaves, leaf detachment force (break strength), activity of abscission-related enzymes, and expression of genes encoding the enzymes in different cotton tissues were determined under greenhouse and/or field conditions. We also estimated the transcript levels of two hydrolytic enzyme genes (*GhCEL1* and *GhPG*) and one ethylene biosynthesis enzyme gene (*GhACS*) in leaf, petiole and leaf abscission zone as well as during leaf abscission. Finally, we determined boll opening, seedcotton yield and seed quality to elucidate whether and how COR affects cotton boll ripening and seed development.

## Materials and Methods

### Plant Material and Coronatine Preparation

The cotton cultivar, Guoxin 3 (GX 3), was selected for the experiment. Seeds of GX 3 were provided by Guoxin Corporation, China. Standard coronatine was provided by Carol L. Bender, Oklahoma State University, Stillwater, OK, USA. The coronatine was prepared as described in Palmer and Bender [45].

### Experiment 1

Seeds of GX 3 were sown in 28 cm diameter pots maintained in a glasshouse under controlled temperature (30±3°C) for about 2 months until the 7^th^ true leaf stage which was approximately 35 days after sowing. At this growth stage, 300 mg L^−1^ COR and TDZ solution were applied evenly to the 7^th^ leaves of ten randomly selected plants at a rate of 1 ml per leaf. Distilled water was similarly applied to the 7^th^ leaves of another ten randomly selected plants as a control. The leaf abscission zone (AZ) was sampled after COR treatment for observation under the electron microscope. Break strength and abscission-related gene expression were determined.

### Experiment 2

Seeds of GX 3 were sown in the field on 29 April 2010 and 27 April 2011. The experimental unit consisted of four rows, 6.5 m long and 0.9 m apart. A randomized complete block design with three replications was used each year. The thidiazuron (TDZ) and coronatine (COR) concentration was 300 mg L^−1^, each applied at 225 L ha^−1^. All treatments were applied during 45–50% boll opening in late September. Break strength, defoliation and ripening effects, cotton yield, and seed quality were examined. Leaf abscission zones (1–2 mm on either side of fracture plane) and other tissues, including leaf blade, petiole and boll crust were harvested, frozen in liquid nitrogen and stored at −80°C for the analysis of hydrolytic enzymes and ACC activities in 2011.

### Scanning Electron Microscopy of Leaf Abscission Zone (AZ)

For the electron microscopy observations, the abscission zones of the 7^th^ leaf were fixed in 4% (v: v) glutaraldehyde in 0.5 mol L^−1^ potassium phosphate buffer (pH 7.4) for 4 h at 25°C, rinsed four times in buffer, and then dehydrated in ethanol through a series of increasing concentrations. Sputter coated sections were then examined at different magnifications with Hitachi S-3400N scanning electron microscope (Hitachi, Japan).

### Determination of Break Strength

Break strength was measured as the force necessary to cause the petiole to separate from the stem across the abscission zone according to Malladi and Burns [33]. This measurement was used as an indicator of the progressive weakening of the tissue in this area at different time points. Petioles from uppermost 3 or 4 nodes were clamped to a digital force gauge (Tayasaf Corporation, China) and force was applied mechanically to the stem. The force at which separation occurred was recorded as the break strength. Petioles from ten randomly selected plants per replicate were used for each treatment. Break strength values obtained were recorded to compute the average break strength per node. Each treatment was repeated three times.

### Hydrolytic Enzyme and ACC Activities

Tissues were pulverized in a mortar under liquid nitrogen and the powder resuspended in extraction buffer (100 mM Tris–HCl, 0.5% PVPP, 10 mM MgCl_2_,10 mM NaHCO_3_, 10 mM DTT, 0.15 mM PMSF, 0.3% (w: v) X-100 Triton and 0.03% sodium azide). The homogenised liquid was filtered through a nylon mesh and centrifuged at 20 000 g for 20 min. The supernatant was dialysed for 16 h at 4°C in extraction buffer diluted 1∶9 (v: v) in water. The samples were then frozen until used.

The extracts were assayed to determine cellulase (CEL) and polygalacturonase (PG) activities, by the viscosity method [46]. A unit of enzyme was expressed as specific activity (U mg^−1^ protein), being the reciprocal of the time in hours to obtain the 50% viscosity loss ×10^3^.

ACC contents were analysed as previously described in Yuan et al. [31]. Tissues were ground to a fine powder in liquid nitrogen using a prechilled mortar and pestle. The powdered tissue was transferred to a centrifuge tube and 10 ml of 80% ethanol was added. The homogenate was centrifuged at 10 000 g for 30 min after incubating the powdered tissue in ethanol at 65°C for 15 min. The residue was reextracted in 10 ml of 80% ethanol at 65°C for 15 min. The supernatants were combined and dried under vacuum. The dry pellet was dissolved in 1 ml of water and extracted once with an equal volume of chloroform. The aqueous phase was collected by centrifugation, dried under vacuum, and redissolved in 0.7 ml of water.

### Quantitative Real-time Polymerase Chain Reaction (qRT-PCR) Analysis

Total RNA was extracted from the leaf abscission zone, leaf blade and petiole using Trizol according to the supplier’s recommendation. Residual DNA was removed with a purifying column. One microgram of total RNA was reverse transcribed using 0.5 µg of oligo(dT) 18 (Invitrogen) and 200 units of Superscript II (Invitrogen) following the supplier’s recommendation. On the basis of expressed sequence tag (EST) sequences, the gene-specific primers were designed and used for amplification [47].

The PCR amplification was performed with gene-specific primers. Primer sequences were designed as follows: *GhCEL1*, forward primer 5′-TTATGGAGAGGTGGGCGATGGT-3′ and reverse primer 5′-CGGATTGCTTGGGTCTTTCTTGT-3′; *GhPG*, forward primer 5′-CACTGCGGCATATGTGTCTAA-3′ and reverse primer 5′-CCTCCCTGCCATGTTTTTATT-3′; *GhACS*, forward primer 5′-GGACTTGTGGCGAGTGATTATC-3′ and reverse primer 5′-AAGCAAACCCTGAACCAACC-3′. The *GhUBQ* gene was used as an internal control to normalize small differences in template amounts with the forward primer 5′-AAGAAGAAGACCTACACCAAGCC-3′ and the reverse primer 5′-GCCCACACTTACCGCAATA-3′.

An Applied Biosystems 7500 Fast Real-Time PCR System (Applied Biosystems, USA) was used for quantitative real-time PCR analyses. Analysis was performed on 1 µl of diluted cDNA in a final reaction volume of 20 µl using the SYBR® Green PCR Master Mix (Applied Biosystems). The PCR conditions consisted of denaturation at 95°C for 3 min, followed by 40 cycles of denaturation at 95°C for 30 s, annealing at 62°C for 30 s, extension at 72°C for 30 s, and a final elongation step of 7 min at 72°C. Primer concentration was optimized and primer validation was performed to enable relative gene expression analysis using the ΔΔCt method [48].

### Defoliation and Boll Opening

Prior to treatment application, 10 plants were randomly tagged from two rows at the center of each plot to count the number of leaves on each plant. The number of leaves was counted again 21 days after treatment (DAT) on the same tagged plants. Defoliation percentage was calculated by equation (1). Opened bolls were determined 21 DAT on the same 10 plants tagged. Bolls on each plant were examined and recorded as either opened or closed and boll opening percentage was calculated by equation (2).
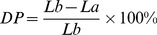
(1)




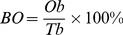
(2)


Where *DP* = Defoliation percentage; *Lb* = Number of leaves before treatment; *La* = Number of leaves at 21 days after treatment; *BO* = Boll opening percentage; *Ob* = Open bolls; *Tb* = Total number of bolls.

### Yield and Seed Quality

Each year, plants from the central two rows in each plot were harvested by hand two times. The first harvest was 21 DAT. The final harvest occurred on 28 October in 2010 and 2 November 2011, about 2 weeks after the first harvest. Seed cotton from each plot was weighed, and subsamples (∼1 kg) were collected, air-dried, and ginned on a 10-saw, hand-fed laboratory gin. Ginning percentage was determined after ginning. Boll weights were determined from 20 randomly selected plants in the central two rows from each plot at first harvest. Seed quality parameters such as seed index (fresh weight per 100 seeds (g)) and germination percentage were determined.

### Statistical Analysis

The experimental data were subjected to an analysis of variance and treatment means were compared using the least significant difference (LSD) at the 5% probability level.

## Results

### Changes in Phenotypic and Anatomical Features of Leaves during Abscission Induced by Coronatine

**Figure 1:**
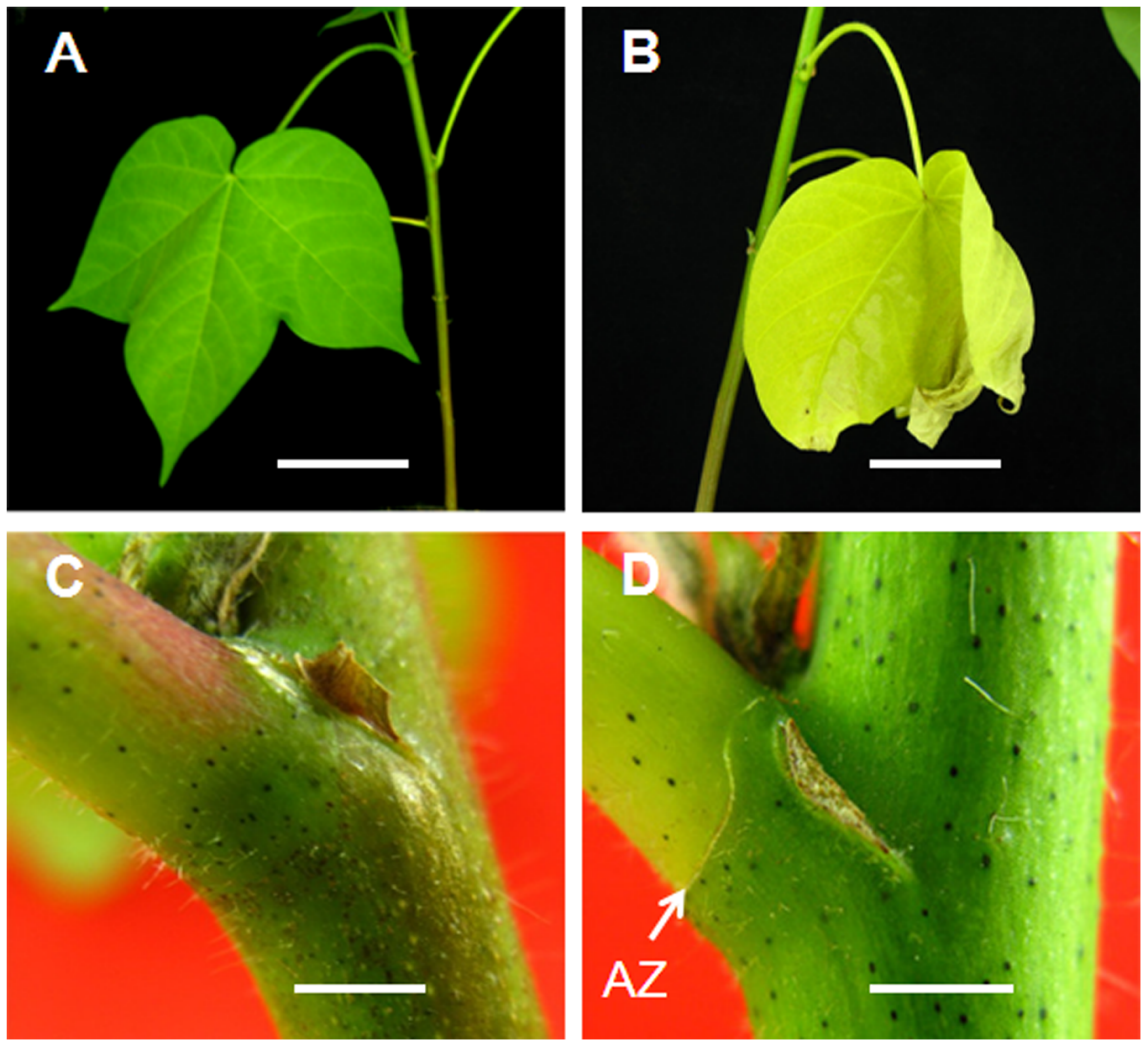
Phenotypic changes in the leaf blade and petiole of coronatine (COR) and water (Control) treated plants. **A** and **C** are phenotypes for 14 d distilled water treated materials, **B** and **D** are phenotypes for 14 d COR treated materials. AZ: leaf abscission zone. *Bar*: **A**, **B** 4 cm; **C**, **D** 3 mm.

**Figure 2:**
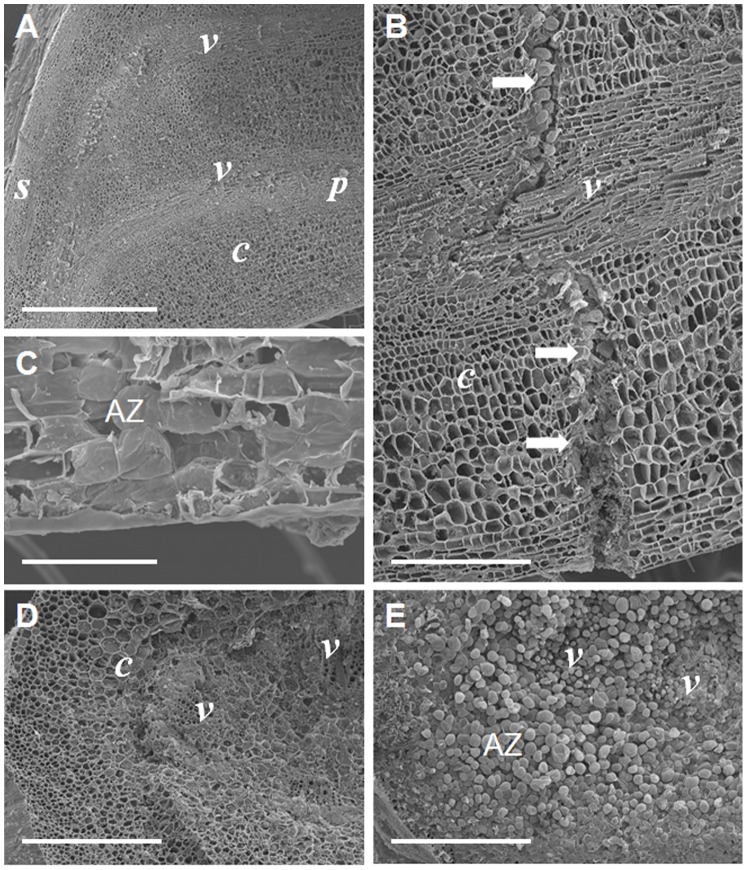
Scanning electron micrograph of cells at the petiole and stem juncture, abscission zone (A, B, C), and fracture plane (D, E) of the cotton leaf abscission zone. **A** and **D** are micrographs of 14 d distilled water treated materials, **B**, **C** and **E** are micrographs of 14 d COR treated materials. *s*: stem, *v*: vascular bundles, *c*: cortex, *p*: petiole, AZ: leaf abscission zone. *Bar*: **A** 1 mm; **B** 400 µm; **C** 100 µm; **D**, **E** 500 µm.

Abscission was accelerated when 300 mg L^−1^ coronatine (COR) solution was administered to cotton leaves (Fig. 1). Disassembly of cell walls in the leaf abscission zone (AZ) should lead to altered anatomical features in this separation layer. The AZs of plants treated with COR and their control were examined under scanning microscopy in order to elucidate the anatomical alterations in AZs (Fig. 2). Compact, well-organized and pentagonal cells were observed on the petiole and stem junction (Fig. 2 A, D) of control plants 14 d after treatment with water; in the COR treated plants, cells of the abscission zone became differentiated and formed (Fig. 2 B, C, E). The treated cells appeared to be elongated and disorganized with a thin cell wall compared to the control.

### Changes in Break Strength of AZ during Leaf Abscission Induced by COR and TDZ

**Figure 3:**
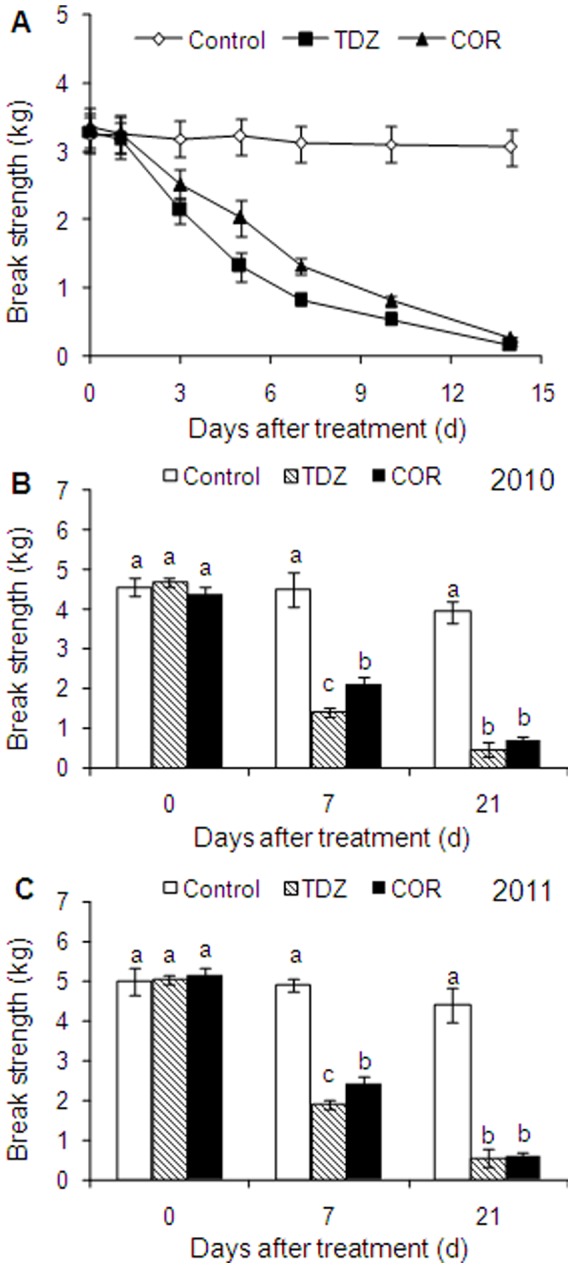
Changes in break strength in abscission zone and adjacent cells treated with water (Control), thidiazuron (TDZ) and coronatine (COR) under glasshouse (A) and field (B, C) conditions. Each value represents the mean ± SE of three replicates. Bars with the same letters are not significantly different.

A significant decrease in break strength was observed in TDZ- and COR-treated plants (Fig. 3A). Although break strength in COR treatment was higher than that in TDZ treatment at 7 DAT, no difference was observed between both treatments at 21 DAT under field conditions (Fig. 3B, C). The break strength in TDZ and COR treatments decreased by approximately 87% at 21 DAT in both 2010 and 2011.

### Changes in Relative Expression of *GhCEL1*, *GhPG* and *GhACS* during Leaf Abscission Induced by COR and TDZ

**Figure 4:**
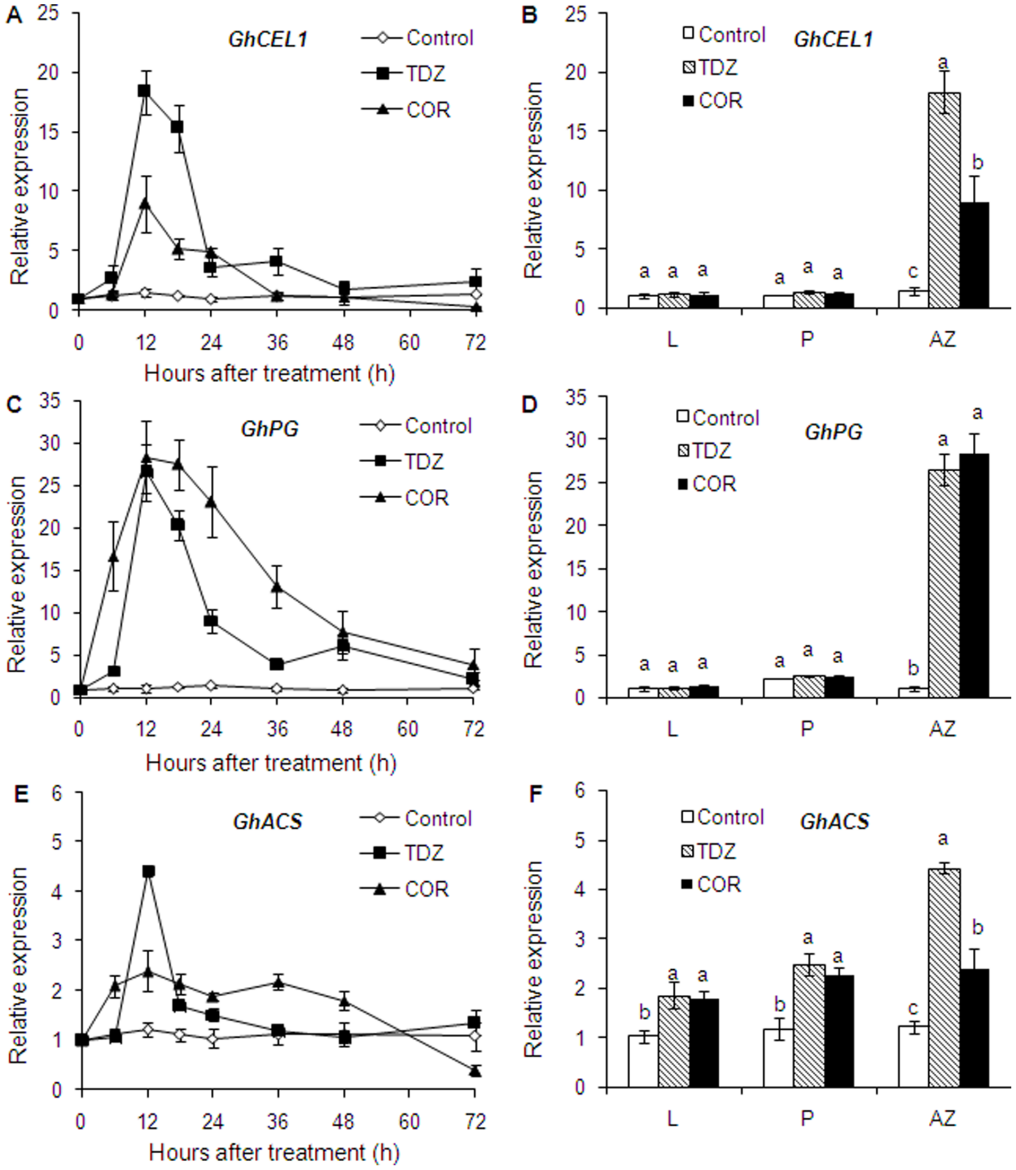
Temporal changes in the relative expression of *GhCEL1*, *GhPG* and *GhACS* in AZ treated with water (Control), thidiazuron (TDZ) and coronatine (COR), and signalling pathways in various cotton tissues at 12 hours after treatment. L: leaf; P: petiole; AZ: leaf abscission zone. Each value represents the mean ± SE of three replicates. Bars with the same letters are not significantly different.

To determine the mechanism of COR induced leaf abscission, we analyzed the expression patterns of several abscission-related genes. Elevated transcripts of *GhCEL1*, *GhPG* and *GhACS* were observed in AZs treated with COR and TDZ (Fig. 4A, C, E). The relative expression of *GhCEL1* and *GhACS* in TDZ treated plants was approximately twice as much as in plants treated with COR for 12 h. However, prolonged expression of *GhPG* and *GhACS* was detected in COR treatment in comparison to TDZ treatment.

Expressions of *GhCEL1*, *GhPG* and *GhACS* were also observed in other tissues such as leaf and petiole at 12 h (Fig. 4B, D, F). No significant effects of TDZ and COR treatments were observed for *GhCEL1* and *GhPG* expression in any tissues other than the leaf abscission zone. A substantial increase in *GhACS* expression was observed in leaf and petiole following TDZ and COR treatment.

### Changes in Activities of Cellulase (CEL), Polygalacturonase (PG) and ACC during Leaf Abscission Induced by COR and TDZ

**Figure 5:**
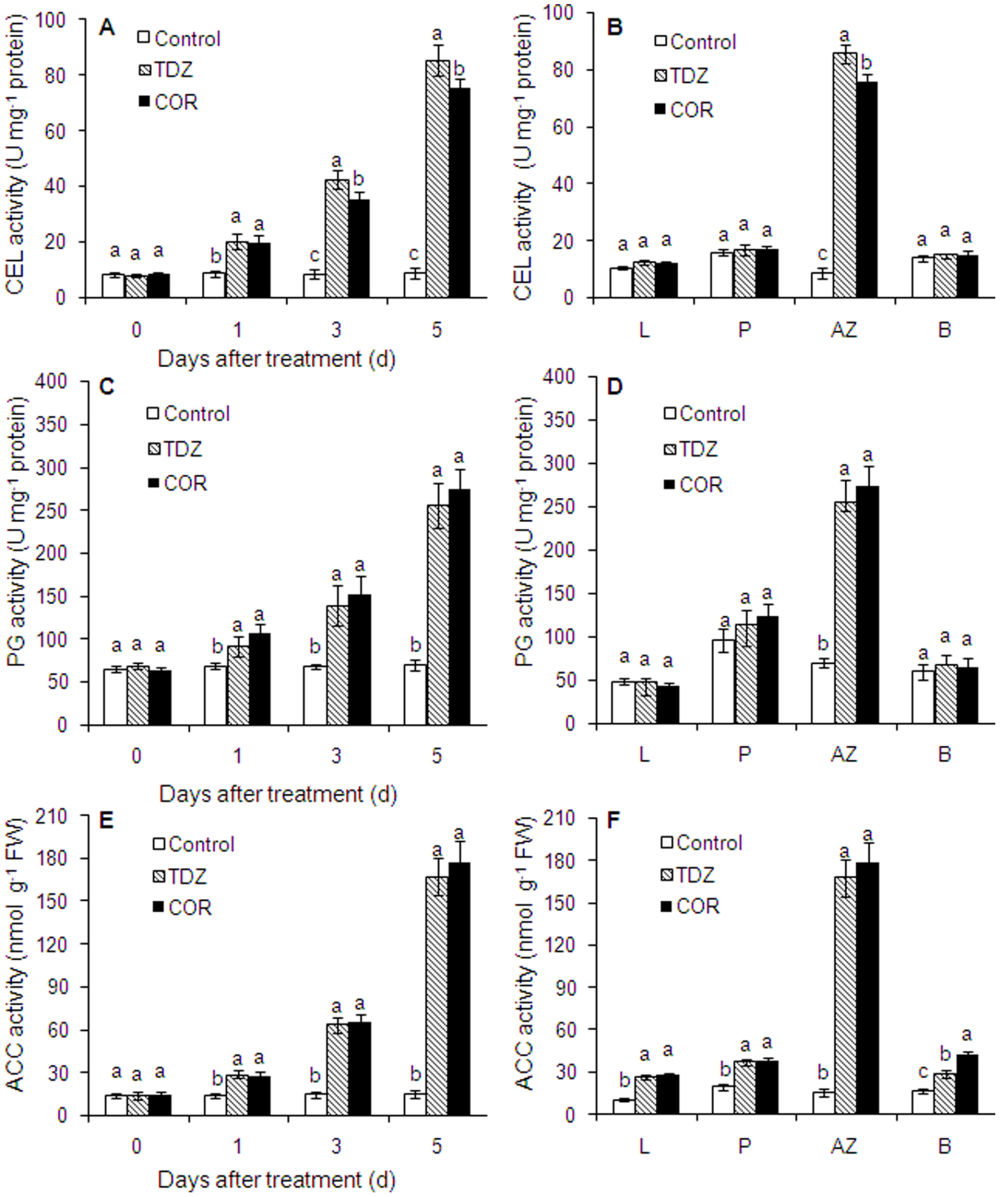
Temporal changes in the activity of cellulase (CEL), polygalacturonase (PG) and ACC in AZ treated with water (Control), thidiazuron (TDZ) and coronatine (COR). Different tissues from Control, TDZ or COR treated plants for 5: leaf; P: petiole; AZ: leaf abscission zone; B: boll crust. Each value represents the mean ± SE of three replicates. Bars with the same letters are not significantly different.

The activities of CEL, PG and ACC in different tissues and AZ during TDZ and COR induced abscission are shown in Fig. 5. There was a continuous increase in the activities of the three enzymes in AZs under TDZ and COR treatments (Fig. 5A, C, E). A 4.9- and 9.7-fold increase in CEL activity was observed in the AZs of TDZ treated plants at 3 and 5 DAT. Similarly a continuous increase (4.1- and 8.6-fold) in cellulase activity was observed in AZs of COR treated plants at 3 and 5 DAT, respectively. A substantial increase in ACC and PG activities were observed after TDZ and COR treatment although no difference was observed between these two treatments in each enzyme.

The CEL, PG and ACC activities were also observed in other tissues such as the leaf, petiole, and boll crust at 5 DAT (Fig. 5B, D, F). No significant effects of TDZ and COR treatments were observed on CEL and PG activities in any tissue other than the leaf abscission zone. However, a substantial increase in ACC activity was observed in petiole, leaf abscission zone, and boll crust after TDZ and COR treatment. In addition, a 50.1% increase in ACC activity of COR treated boll crust relative to the treatment of TDZ was observed.

### Changes in Defoliation and Boll Opening of Cotton Treated with COR and TDZ

**Figure 6:**
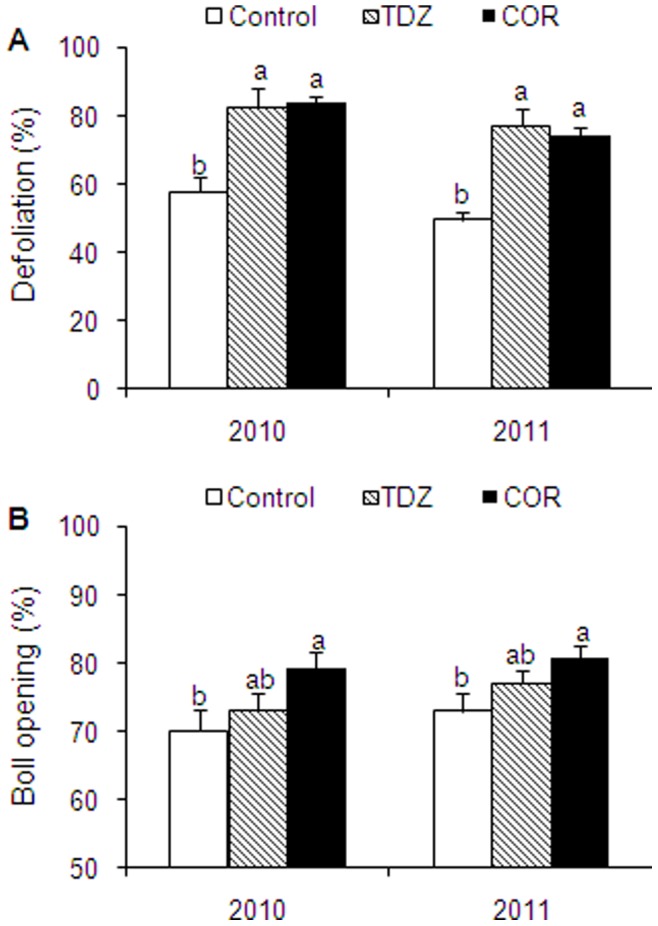
Effect of Thidiazuron (TDZ) and coronatine (COR) on defoliation and boll opening at 21 days after treatment in 2010 and 2011. Each value represents the mean ± SE of three replicates. Bars with the same letters are not significantly different.

Defoliation was increased by TDZ and COR treatments at 21 DAT in both experiments of 2010 and 2011 (Fig. 6). Whereas the defoliation percentage for the control plants averaged 54.2% in 2010 and 2011, it averaged above 80.0% for the TDZ and COR treatments. Boll opening increased by about 8.3% in the COR treatment but not was significantly increased in the TDZ treatment.

### Changes in Seedcotton Yield and Seed Quality following Treatment with COR and TDZ

First harvest yield and first harvest percentage significantly increased in the COR treatments, but not in TDZ treatment except the first harvest yield in 2010 (Table 1). Although the difference between COR and TDZ treatments was not significant, a trend was noticed that COR treatment was more effective in increasing the first harvest yield. For the controls, the first harvest yield ranged from 70.8 to 77.1% of total yield. This percentage increased to about 83.4 to 87.3% of the total yield in the COR treatment. Boll weight, ginning percentage, seed index, and germination percentage were not influenced by COR treatment.

**Table 1:** Effect of Thidiazuron (TDZ) and coronatine (COR) on seedcotton yield and seed quality in 2010 and 2011.

Year	Treatment	1^st^ harvestyield (kg ha^−1^)	Totalyield (kg ha^−1^)	1^st^ harvestpercentage (%)	Bollweight (g)	Ginningpercentage (%)	Seedindex (g)	Germinationpercentage (%)
2010	Control	2599.8^b^ *	3650.8^a^	71.2^b^	5.67^a^	39.1^a^	10.9^a^	91.3^a^
	TDZ	2877.1^a^	3681.4^a^	78.2^ab^	5.40^a^	38.7^a^	10.1^a^	90.2^a^
	COR	3049.9^a^	3703.8^a^	82.6^a^	5.53^a^	39.4^a^	10.8^a^	91.8^a^
2011	Control	3011.9^b^	3925.5^a^	76.7^b^	5.86^a^	38.3^a^	11.3^a^	89.8^a^
	TDZ	3289.1^ab^	3909.4^a^	84.0^ab^	5.83^a^	39.2^a^	10.7^a^	88.7^a^
	COR	3461.8^a^	3978.5^a^	87.1^a^	5.96^a^	38.7^a^	10.2^a^	90.3^a^

*For each factor, means within the same column followed by different letters differ significantly (P≤0.05).

## Discussion

Appropriate and safe abscission chemicals will improve timing and facilitate harvest of cotton. In this study, we demonstrated that the phytotoxin, coronatine induced leaf abscission during cotton defoliation. Abscission occurs in an anatomically distinct cell layer known as the abscission zone (AZ) [49]. The abscission zone is defined as the region at base of abscising organs through which abscission eventually occurs. The anatomy of abscission is important for understanding the biology of a given plant species since form and structure comprise an appropriate starting point for potential functional comparisons between botanically distinct organs [50], [51]. Our data showed that abscission was accelerated when COR solution was applied to cotton leaves at 300 mg L^−1^. Disassembly of cell walls in the AZ should lead to alteration in anatomical structures in this separation layer. Leaf abscission zone cells were examined by scanning microscopy to elucidate the anatomic mechanisms of COR induced abscission in cotton leaves. After 14 d treatment with COR, the cells of AZ became elongated and disorganized, and the cell wall became thinner than that of control plants. It was also observed that COR alone could initiate the abscission process. The enlarged cells of the abscission zone seemed to have undergone a programmed cell death or physical dissolution in which the cells lost integrity. These results are consistent with a previous argument that while the abscission zone consists of several layers of cells across the petiole, the vascular bundles remain intact, allowing transportation of water and nutrients in and out of leaves [52].

The COR treated leaf abscission zone showed a greater decrease in break strength than the control, suggested that the COR effect was over and above the wounding effect. Similar observations have been made in citrus fruit abscission zones in which the break strength decreased after COR treatment [28]. The break strength under COR treatment was higher than that under TDZ treatment at 7 DAT, but not at 21 DAT. This suggests that leaf abscission induced by COR is relatively moderate, and could allow timely nutrient transport from cotton leaves to bolls.

High synthesis and activities of cell wall hydrolases, including β-1, 4-glucanase or cellulase (CEL) and polygalacturonase (PG), were observed in most abscising events which could be responsible for the degradation of middle lamella and the loosening of primary cell wall in separation layers [29], [36]. Mishra et al. analyzed the effects of some phytohormones such as ABA and IAA on cellulase and PG activities of cotton leaf explants. The increase in cellulase and PG activity in the LAZ of the ABA treated explants relative to control explants suggested the roles of ABA in this increment. The process of leaf abscission in cotton was associated with higher biosynthesis of ethylene in abscission zones along with elevated levels of cellulase activity [3]. In the current study, both COR and TDZ induced elevated transcripts of *GhCEL1* and *GhPG* in AZs, but not in leaf blades and petioles (Fig. 4). No differences were observed in the maximum expression of *GhPG* and PG activity between COR and TDZ treatments. *GhCEL1* maximum expression and CEL activity in AZ treated with TDZ were higher than those in plants treated with COR. This resulted to a smaller reduction in break strength under COR treatment than under TDZ treatment (Fig. 3). Nevertheless, for the final levels of break strength and defoliation, there were no differences between COR and TDZ treatments (Fig. 3 and 6).

Ethylene production and ACC accumulation increased in abscised tissues or organs such as leaves and fruits treated with ethephon or other exogenous chemicals [31], [53]. Increased ethylene biosynthesis through over-expression of ACS leads to premature flower abscission, while a block in ethylene perception in the never ripe (nr) mutant delays petal abscission in tomato [37], [54]. ACS1 is mainly involved in system II-like ethylene biosynthesis in citrus. Increased expression of ACS1 in mature fruit and leaf abscission zones was associated with ethephon-induced abscission [31]. In this work, *GhACS* in AZs was upregulated by TDZ and COR treatment whether in leaf blades or petioles (Fig. 4). A substantial increase in ACC activity was observed in petiole, leaf abscission zone, and boll crust after TDZ and COR treatment. Although *GhACS* expression in AZ treated with TDZ was approximately 2.0-fold higher than that in plants treated with COR for 12 h, prolonged expression time of *GhACS* was observed in COR treatment compared with TDZ treatment. Thus, no difference in ACC activity was noted between COR and TDZ treatment. The application of ethylene induced cotton defoliation and increased the percentage of open bolls [55], [56]. The increased ACC activity of COR treated boll crust relative to that of TDZ indicated that COR can induce more ethylene in boll crust. Thus, it is beneficial to increasing the percentage of open bolls.

Abscission-inducing chemicals can increase machine harvest efficiency, improving lodging, and reducing the time from defoliation to harvest. Numerous studies have focused on effects of exogenous chemicals on defoliation and boll opening [4], [57], [58]. Defoliants such as TDZ had greater defoliation effects but did not directly influence boll opening [5], [59]. In this study, it was found that COR induced both defoliation and boll opening. The higher boll opening under COR treatment might have been associated with increased ACC activity in boll crust. Although approximately 85% abscission and 80% boll opening were observed for COR treatment, defoliation and boll opening were lower than those reported in Gwathmey and Hayes [59]. Further studies on both dosage and application timing of COR are necessary in cotton to optimize its use as a harvest aid chemical.

Defoliation allows producers to harvest earlier than allowing crops to mature naturally. However, the practice with defoliation may reduce yield and alter fiber quality if the application of harvest aids is premature (e.g., prior to 60% open bolls) [2]. Defoliation may increase the total harvest yield only if defoliant or boll opener increases the number of open bolls at harvest. On the other hand, it may reduce boll weight by opening small bolls prematurely and further decrease yield [60]. Recent evidence suggests that defoliation could be initiated before 60% open bolls if fruiting is compact (i.e., fruit set over eight to ten nodes); however, a crop with extended fruiting may require delayed defoliation to achieve maximum yields [61]. Although our study was conducted with a relatively early application of abscission chemicals (45–50% open bolls), the total seedcotton yield, boll weight, lint percentage, seed quality, and fiber quality (data not shown) were unaffected by either COR or TDZ treatment. In addition, the first harvest yield and first harvest percentage were significantly increased by COR. Although the difference between COR and TDZ treatments was not significant, COR was more effective in increasing the first harvest yield than TDZ.

In conclusion, this work provides structural, biochemical and molecular evidence that the phytotoxin, coronatine affects cotton abscission by increasing *GhCEL1*, *GhPG* and *GhACS* expression, and activity of hydrolytic enzymes such as CEL and PG as well as ACC accumulation in AZ through mechanisms dissimilar to those of TDZ. In particular, the greater increase in ACC activity of COR treated boll crust suggests that COR has better ripening effect than TDZ. It is possible that COR can induce both defoliation and boll ripening in cotton without adverse effects on yield and seed development.
